# Changes in the Level of hs-CRP in the Blood and Morphometric Parameters of Tissues Following Implantation of Polypropylene

**DOI:** 10.3390/ijms26041419

**Published:** 2025-02-07

**Authors:** Igor A. Eisenach, Galina A. Lapii, Alexandra K. Uzyumova, Elena L. Lushnikova, Victor S. Ovchinnikov, Anastasia O. Solovieva, Orlan V. Oorzhak, Alexey V. Kuznetsov

**Affiliations:** 1 Institute of Molecular Pathology and Pathomorphology, Federal Research Center for Fundamental and Translational Medicine, Novosibirsk 630117, Russia; galapii@frcftm.ru (G.A.L.); apichigina@yandex.ru (A.K.U.); ellushnikova@frcftm.ru (E.L.L.); 2 Research Institute of Clinical and Experimental Lymрhology–Branch of the Institute of Cytology and Genetics, Siberian Branch of Russian Academy of Sciences, Novosibirsk 630060, Russia; ovch.v.s@mail.ru (V.S.O.); solovevaao@yandex.ru (A.O.S.); 3 State Autonomous Healthcare Institution “Kuzbass Regional Clinical Hospital Named after S.V. Belyaeva”, Kemerovo 650066, Russia; oorjakov@mail.ru; 4 Federal State Budgetary Educational Institution of Higher Education Novosibirsk State Medical University Ministry of Health of Russia, Novosibirsk 630091, Russia; 1xo2788353@mail.ru

**Keywords:** polypropylene, implant, tissue reaction, inflammation, bioinertness, highly sensitive C-reactive protein (hs-CRP)

## Abstract

In recent decades, the use of polypropylene meshes has been the gold standard in the surgical treatment of muscular corset failure. However, the reasons behind the low percentage of complications and recurrences remain controversial. Tissue hyperreactivity and the immune response to polypropylene may be contributing factors. Measurements of the level of hs-CRP (highly sensitive C-reactive protein) in the blood and morphometric studies of tissues around the implant were carried out for three months after the installation of polypropylene implants in 53 laboratory rats. The research results confirmed the good biocompatibility of polypropylene and the formation of full-fledged connective tissue around polypropylene three months after the installation of the material. The level of hs-CRP in the blood increased slightly, without significant differences, but in some animals, there was a sharp increase in this indicator at 3 months. Such results may indicate the development of hyperreactivity to the implantation of a synthetic material and, with other accompanying factors, lead to the development of complications both at the local tissue and general immune levels.

## 1. Introduction

Extensive surgical experience indicates that polypropylene is bioinert, takes well to tissues, does not trigger allergic reactions, and is extremely rarely rejected [1]. Reported complications are also uncommon, including seroma, infection, necrosis, fibrosis, and others [2,3]. However, their frequency increased with the introduction of polypropylene in urogynecological practice for stress urinary incontinence and pelvic organ prolapse [4,5]. One of the most common complications of these operations, vaginal erosion, is not serious, but can diminish surgical effectiveness and the quality of life of patients [3,6,7]. In the last decade, practicing surgeons have been asking themselves the question: is the implantation of polypropylene material limited to only a local tissue reaction, or can it lead to the development of adaptive and reactive processes to foreign material throughout the body over time [8,9]? The syndrome of autoimmune inflammation induced by adjuvants (ASIA), as described in clinical practice recently, supports further investigation into this question [10,11]. The literature describes attempts to monitor the body’s response to polypropylene implants in patients by tracking C-reactive protein (CRP), an inflammation marker. An increase in the level of CRP may be a consequence of the activity of inflammatory mediators in the tissues operated on the implant—interleukins, cytokines, and others [12,13]. Studies did not give a clear answer, since the presence of purulent processes in the peri-implantation tissues cannot be excluded, which can lead to the development of a general inflammatory response and an increase in the C-reactive protein (CRP) [14].

In such cases, a morphological study of the peri-implantation tissues in parallel with the level of hs-CRP is of interest. Given the small size of the area of the polypropylene meshes in comparison with the area of the patient’s body, it may be more appropriate to use hs-CRP. Histological studies on patients are invariably complex. Therefore, the study of the inflammatory response in the tissues of experimental animals in parallel with the study of the dynamics of inflammatory markers in the blood on the implantation of polypropylene in remote periods in the experiment is relevant, which became the aim of our study. The objective of this research is to study the inflammatory response to synthetic material by the dynamics of morphometric indicators in tissues and the level of highly sensitive C-reactive protein in the blood of experimental rats after the implantation of polypropylene material.

## 2. Results

### 2.1. Tissue Response to Polypropylene (Macropreparation, Morphometry)

**Figure 1:**
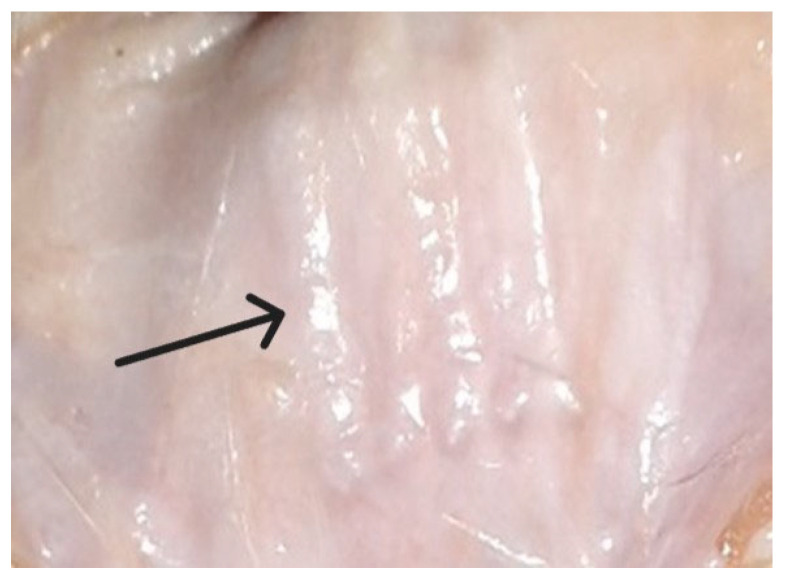
Polypropylene with clear boundaries in tissues with a cellular structure (to the right of the arrow), 3 months after implantation (macropreparation).

In group I of the animals, no visible signs of pathological processes were detected after the installation of the polypropylene material. No suppuration or rejection of the implanted material was observed in any animal. Figure 1 shows the implanted polypropylene material at the third month of the experiment.

**Figure 2:**
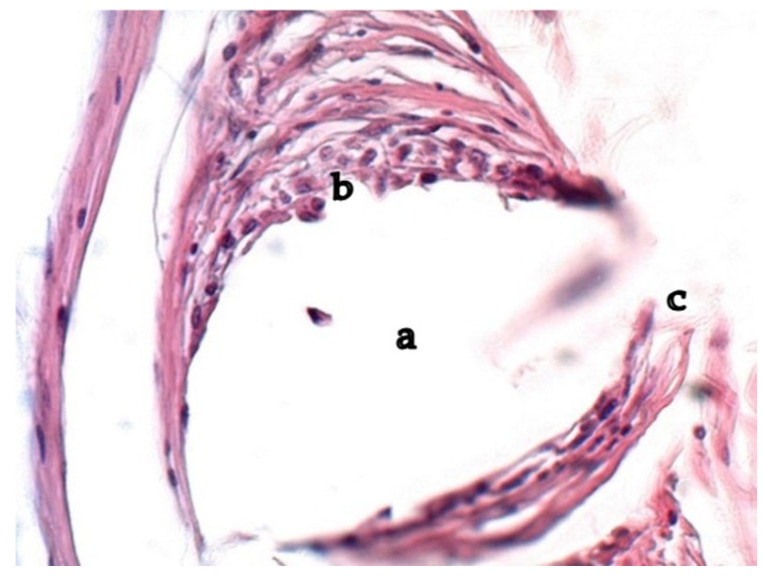
Infiltrate around polypropylene, 1 month of the experiment. Cell clusters and forming collagen fibers near the fiber surface. a—polypropylene, b—FBGC, leukocytes and lymphocytes, c—fibroblasts and collagen fibers. Hematoxylin and eosin staining. Magnification 200.

The macropreparations visualized the cellular structure and boundaries of the material at all observation periods; hemorrhages or foci of infiltration along the perimeter and in the thickness of the matrix were not identified. The size and structure of the material did not change. As early as the 14th day, a white connective tissue capsule was identified around the implant, which was confirmed by micropreparations in the form of a zone of cellular infiltration from resident and non-resident cells around each fiber. Leukocytes, macrophages and lymphocytes, as well as fibroblasts and fibrocytes, migrated en masse from the vessels to the alteration zone. Figure 2 shows a micropreparation one month after implantation when a restrictive granulation ridge was formed around the synthetic fibers of the mesh.

Fibroblasts were detected in the infiltrate around the polypropylene fibers, sometimes spreading parallel to the surface in a thin layer with collagen fibers, forming a ring shape surrounding the synthetic polypropylene fiber. A large number of leukocytes and lymphocytes were detected between fibroblasts and collagen fibers, and mast cells were present in small quantities. Single visualized FBGC (foreign body giant cells) were localized either near the foreign body or in the cellular infiltrate. Elastic fibers in the infiltrates around polypropylene were few in number and difficult to visualize. In the infiltrate, a zone of dense cellular formation adjacent to the polypropylene and, at a distance, a zone of loose cellular formations were identified. The number of vessels per unit area decreased, and their area increased sharply at the border of dense and loose connective tissue. Collagen bundles also became more frayed, diffusely scattered over the entire area closer to the loose cellular formation.

**Figure 3:**
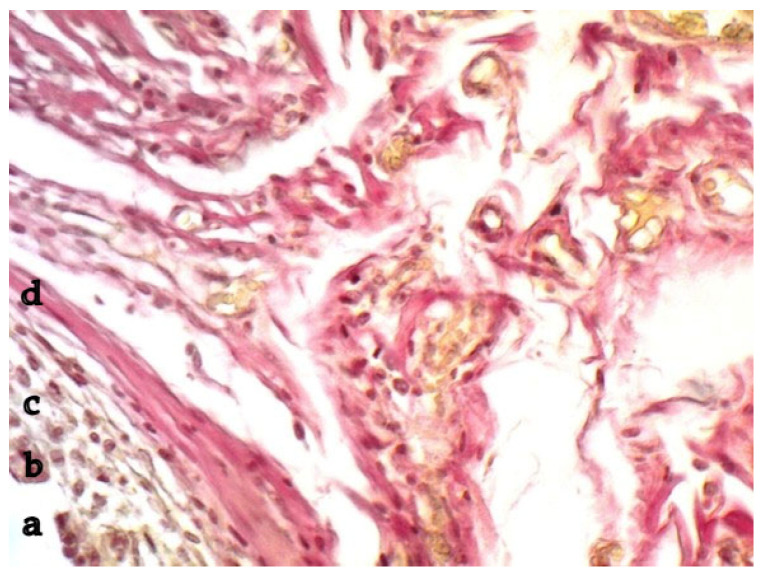
Infiltrate around polypropylene, 2 months of the experiment. Fibroblasts, forming vessels, and a proliferation of thin collagen fiber bundles. a—polypropylene, b—FBGC, c—leukocytes and lymphocytes, d—fibroblasts and collagen fibers. Van Gieson staining. Magnification 200.

Figure 3 shows a micropreparation two months after implantation.

At this time, the inflammatory response had decreased, and the vessels were located on the second line from the foreign matter after the resident cells and collagen fibers surrounded by a small number of fibroblasts. Three months after implantation, a full-fledged dense connective tissue was formed around the polypropylene fibers.

The dynamics of the number of cells in the tissues around the implant reflected the inflammatory and fibroplastic processes.

**Figure 4:**
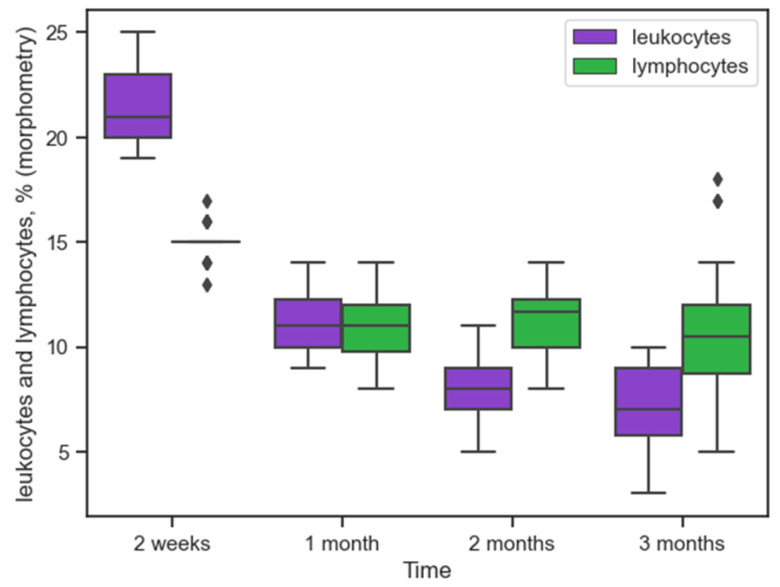
Dynamics of leukocyte and lymphocyte reactions to implanted polypropylene over time (morphometry (%) in tissues), (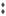 is outlying data).

Figure 4 shows the inflammatory response associated with the migration of lymphocytes, leukocytes.

This response was most pronounced at 14 days, leukocytes 21% [20%; 23%], lymphocytes 15% [15%; 15%], and significantly decreased by the end of the first month, leukocytes 11% [10%; 12.5%], U = 0 (*p* = 0), lymphocytes 11% [9.5%; 12%], U = 3.5 (*p* = 0). Over the next two months, changes in the number of inflammatory cells were not so significant. The median values in the second month for leukocytes 8% [7%; 9%] and lymphocytes 11.7% [10%; 12.5%] did not differ significantly from the values in the third month: 7% [5.5%; 9%], U = 147 (*p* = 0.16), and 10.5% [8.5%; 12%], U = 167 (*p* = 0.38), respectively. Figure 4 shows single points significantly exceeding the quartile range for lymphocytes at three months, indicating an increase in the number of these cells in the inflammatory infiltrate in some animals.

**Figure 5:**
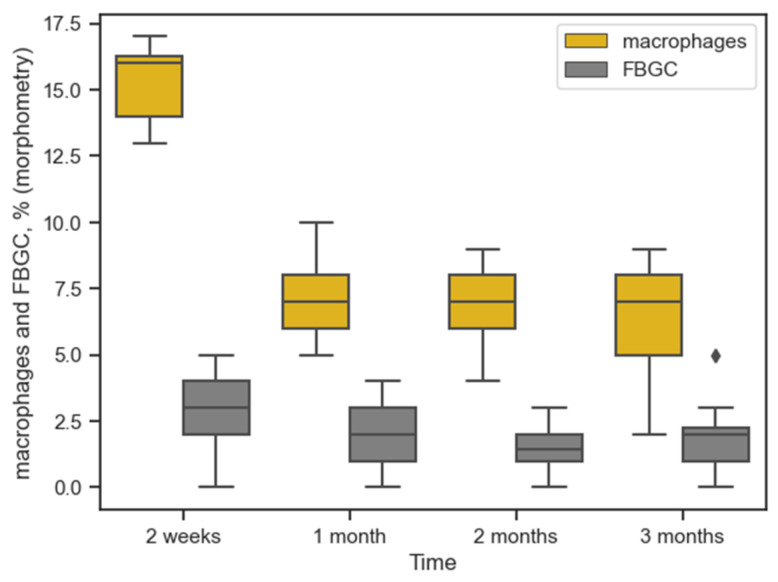
Dynamics of macrophage and FBGC reaction around implanted materials over time (morphometry (%) in tissues), (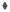 is outlying data).

Figure 5 shows the dynamics of the number of macrophages and FBGC.

**Figure 6:**
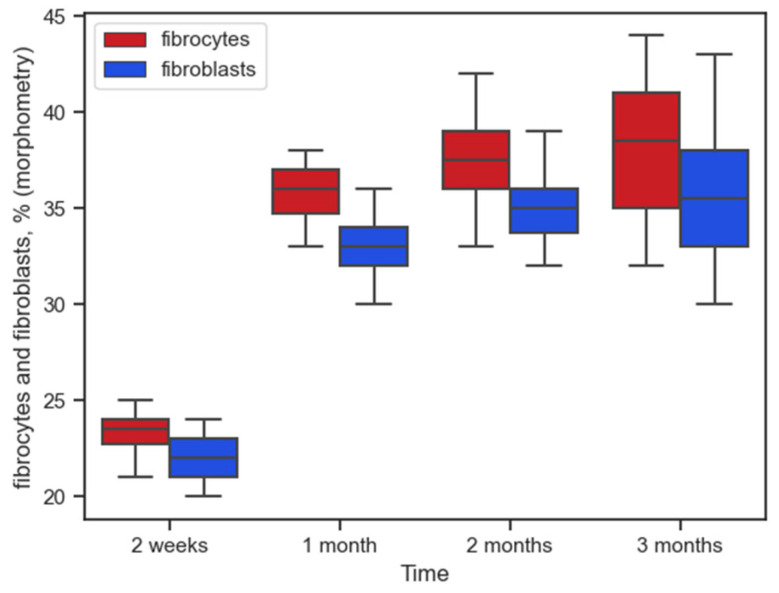
Dynamics of fibrocytic and fibroblast reaction to implanted polypropylene over time (morphometry (%) in tissues).

The maximum number of these cells was also at 14 days: 16% [14%; 16.5%] and 3% [2%; 4%], respectively. In the first month, there was a significant decrease in macrophages to 7% [6%; 8%], U = 0 (*p* = 0). The medians of the number of macrophages did not differ significantly for the first and second months: 7% [6%; 8%] and 7% [6%; 8%], respectively, U = 185 (*p* = 0.69). There was no significant difference in the second and third months either, U = 169 (*p* = 0.42). The amount of FBGC was insignificant at all times and did not change significantly over time. The processes of connective tissue capsule formation in the form of fibroplastic reaction lagged slightly behind the inflammatory one, by approximately one month. The median values for fibrocytes and fibroblasts for the first and second months were still significantly different: fibrocytes 36% [34.5%; 37%] and 37% [36%; 39%], U = 104.5 (*p* = 0.01); fibroblasts 33% [32%; 34%] and 35% [33.5%; 36%], U = 93.5 (*p* = 0.004). The values for these cells for the second and third months were no longer significantly different—U = 171 (*p* = 0.44) and U = 165.5 (*p* = 0.36), respectively. Figure 6 shows the dynamics of the number of these cells.

In accordance with the changes in the cellular and non-cellular components of the infiltrate, the outcome of the response to the implantation of the polypropylene material was the formation of dense, formed connective tissue after three months, with many unidirectionally located mature collagen fibers, while maintaining a weak inflammatory reaction to the foreign synthetic material.

### 2.2. Animal Experiment, Level of hs-CRP in Blood, ELISA (Enzyme-Linked Immunosorbent Assay)

**Figure 7:**
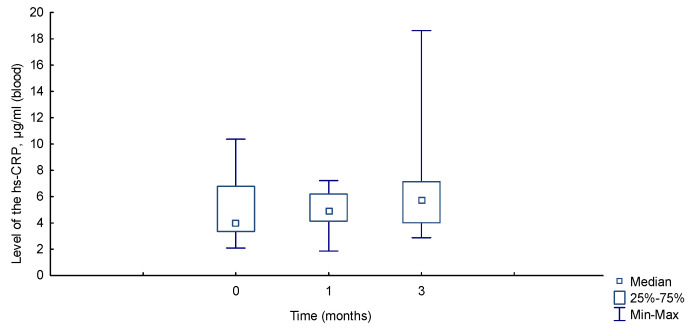
Dynamics of hs-CRP in the blood at different times (μg/mL).

The animals in group II also tolerated the experiment satisfactorily, without manifesting visible pathological processes. Figure 7 shows the dynamics of the level of hs-CRP in the blood of animals.

According to literary sources, the average values of hs-CRP in the group of intact animals not subjected to additional effects were in the range from 4.0 ± 0.4 to 5.71 ± 0.27 mg/L [15,16]. At the initial point of our study, the level of hs-CRP in the blood of intact animals was also within these limits by the average value—4.84 ± 2.23 μg/mL, by the median—3.97 μg/mL [3.36 μg/mL; 6.78 μg/mL]. According to the results, the median level of hs-CRP in the blood of animals increased slightly after one month compared to the initial level, before implantation of the material: 4.92 μg/mL [4.14 μg/mL; 6.2 μg/mL] and 3.97 μg/mL [3.36 μg/mL; 6.78 μg/mL], respectively, but without a significant difference U = 191 (*p* = 0.47).

After three months, this value was even greater than after one month: 5.69 μg/mL [4.02 μg/mL; 7.13 μg/mL] and 4.92 μg/mL [4.14 μg/mL; 6.2 μg/mL], respectively—but also without a significant difference, U = 181 (*p* = 0.33).

It is evident from Figure 7 that the amplitudes of deviations in the hs-CRP level have changed significantly, especially upwards, i.e., excesses with deviations from the median upwards were recorded. The following features were recorded during the analysis of the dynamics of specific animals. The animals with the baseline hs-CRP levels before implantation were slightly higher than the median, had the highest hs-CRP levels after one and three months. This could be indicative of hyperreactivity to the implantation of the material in some animals. For example, animal No.7: before surgery—6.75 μg/mL; after one month—6.78 μg/mL; after three months—18.6 μg/mL. There were examples of the opposite dynamics, when the highest hs CRP level before surgery decreased dynamically and was within the average value for the study group at a given time. Animal No.5: before surgery—10.36 μg/mL; after one month—4.3 μg/mL; after three months—3.55 μg/mL.

When comparing the mean values before surgery and after three months: 3.97 μg/mL [3.36 μg/mL; 6.78 μg/mL] and 5.69 μg/mL [4.02 μg/mL; 7.13 μg/mL], respectively, no significant difference was found, U = 155 (*p* = 0.1).

These results may give reason to assume that in some animals, for various reasons, a hyperergic reaction to the polypropylene material may develop after three months, at a time when the inflammatory response in the tissues, according to morphometry, has ended.

## 3. Discussion

Already during the fifties of the twentieth century, surgeons began to make the first attempts to use synthetic polymers in the treatment of muscle failure in humans. One of the first was perforated fluoroplastic. In the seventies, polyester was used, but frequent purulent complications were registered. It was assumed that this polymer is bacteriophilic. Later, this assumption was refuted. Due to its physical characteristics of high wear resistance to physical, chemical, temperature effects, as well as hydrophobicity and lack of degradation in biological tissues, polypropylene has become the most popular material in the surgical treatment of abdominal hernias since the eighties of the twentieth century [17,18,19,20].

According to the literary sources, the use of polypropylene materials in surgical practice to strengthen the muscle frame ends with the formation of connective tissue around the mesh fibers after one to three months [21,22,23].

According to the study conducted, dense connective tissue was formed, taking into account cellular and non-cellular structures formed by the third month after implantation. The cellular composition of peri-implantation tissues changed insignificantly quantitatively two months after surgery. These results allow us to conclude that the inflammation process from the moment of alteration to proliferation and restoration of function was completed after three months and that the short duration of the experiment was sufficient enough to track the tissue response to the implantation of the synthetic material. The dynamics of the increase in the level of hs-CRP by the median value for three months were without significant differences. But, isolated cases of a sharp increase in the level of inflammatory protein three months after surgery give us a reason to suspect hyperreactivity of some subjects to xenomaterial, as well as the need for a longer experiment to track the level of hs-CRP at a later date.

The registered cases of increased lymphocyte counts in peri-implantation tissues may also indicate the development of tissue hyperreactivity to polypropylene (Figure 4). For technical reasons, it was impossible to conduct a study on one group in the experiment with morphometry and a measurement of the hs-CRP level in the blood since the subjects selected for histological examination would not have allowed tracking the dynamics of hs-CRP changes. In this regard, it is impossible to compare tissue response and morphometry data with the level of inflammatory protein in each subject. Such studies may be of practical interest in the future. Of great practical interest may be studies of hs-CRP levels in patient groups. It is generally accepted in the scientific community that violation of surgical technique leads to the development of delayed inflammatory complications [24,25].

For example, it is believed that the development of vaginal erosions after pelvic organ prolapse plastic surgery with polypropylene materials is a consequence of damage to the pubocervical ligament during surgery. The size of such erosions often does not exceed 2–3 cm in diameter without pronounced purulent processes [26,27,28]. It can be assumed that such patients are unlikely to have general inflammatory changes in the blood, including hs-CRP. Therefore, it is of interest to study the level of hs-CRP in patients with such complications in order to study the reaction of the whole body to the polypropylene material. These studies can supplement the information on autoimmune syndrome induced by adjuvants (ASIA) and develop recommendations for preoperative preparation and postoperative management of patients.

## 4. Materials and Methods

Two groups of 32 and 21 laboratory white male Wistar rats, aged 3 months, were implanted with a 1 × 1 cm polypropylene mesh having a surface density of 28 g/m^2^, elongation 51%, breaking tenacity 24 daN under the skin of the back. The experiment used standard material approved in medical surgical practice for the correction of muscle failure.

The studies were conducted in accordance with the Declaration of Helsinki of the World Medical Association on the humane treatment of laboratory animals and the directive of the European Community (Directive 86/609/EEC).

### 4.1. Animal Experiment, Tissue Research, and Morphometry

In group I consisting of 32 laboratory rats, 8 animals were withdrawn from the experiment after 2 weeks, and then after 1, 2, and 3 months. Histological material was collected in the form of a polypropylene with surrounding tissues. After fixing the samples in a 10% solution of neutral formalin and standard processing on a Leica TP1020 (Leica Biosystems, Heidelberg, Germany) histological complex, they were embedded in paraffin blocks. Sections 3–4 μm thick were made on a Leica RM2235 (Leica Biosystems, Heidelberg, Germany) rotary microtome. Paraffin sections were stained with hematoxylin and eosin in a Leica ST5010 apparatus and according to van Gieson to identify collagen fibers. The histological preparations were examined in an Olympus CX 31 (Olympus, Tokyo, Japan) microscope with a Nikon DS-Fi1 digital video camera (Nikon, Tokyo, Japan). In the implantation zones, the formed granuloma was assessed in close proximity to the synthetic material, and the qualitative and quantitative parameters of the cellular and non-cellular components of the connective tissue surrounding the implanted samples were determined. The number of neutrophilic leukocytes, fibrocytes, fibroblasts, lymphocytes, macrophages, and foreign body giant cells (FBGC) was counted. Cells of at least 100 were counted in the field of view at a magnification of 400 times, the number of each type of cells was expressed as a percentage. The median values of the number of cells at different times were compared using the nonparametric U test Mann–Whitney. The analyzed values were measured automatically using the Bio Vision program (4.0, West Medica Produktions- und Handels- GmbH, Wiener Neudor, Austria).

### 4.2. Animal Experiment, Study of hs-CRP, ELISA

In group II consisting of 21 animals, blood was collected from the animals’ tails at three timepoints: on the day of the surgery before implantation of the material; 1 month after implantation of the material; and 3 months after implantation of the material. Blood was collected in test tubes with EDTA (Ethylenediaminetetraacetic acid), followed by its study by the ELISA (Enzyme-linked immunosorbent assay) kit for hs-CRP (Elabscience, Wuhan, China). Spectrophotometry was performed using a Multiscan Sky device (Termo scientific, Singapore). The hs-CRP measurements obtained at different timepoints were compared with each other by the median value to determine any significant difference using the nonparametric U test Mann–Whitney. Statistical analysis of the material and graphical representation were performed using the Statistica (6.4, StatSoft, Palo Alto, CA, USA) and Python 3.2 application software package.

## 5. Conclusions

Animal tissue studies confirmed good biocompatibility of polypropylene. The tissue reaction was characterized by moderate inflammatory, high-quality fibroplastic processes, ending with the formation of dense, well-formed connective tissue by the second month.

A parallel study of the dynamics of hs-CRP also confirmed the bioinertness of polypropylene and the absence of inflammatory signs in animals. A tendency towards a slight increase in its level in the remote periods after implantation of the polypropylene material was revealed but without displaying a significant difference for different periods. The highest level of hs-CRP was registered three months after implantation when the inflammatory tissue reaction to the implanted material was on a downward trend and the number of most inflammatory cells did not change after the first month. During this period, isolated cases of a sharp increase in the level of inflammatory protein were detected, which could be a consequence of the hyperreactivity of individual animals to the implantation of the synthetic material.

According to morphometry data, isolated cases of an increase in the number of lymphocytes were also detected after three months. Individual hyperreactivity to polypropylene may be the cause of complications both at the tissue and general immune level. Hs-CRP can be recommended for assessing the adaptation processes both in tissues and the whole organism after using polypropylene in surgical practice.

## Data Availability

The data that support the findings of this study are available from the corresponding author upon reasonable request.

## Abbreviations

The following abbreviations are used in this manuscript:

Hs-CRP	Highly sensitive C-reactive protein
ASIA	Autoimmune syndrome induced by adjuvants
CRP	C-reactive protein
ELISA	Enzyme-linked immunosorbent assay
EDTA	Ethylenediaminetetraacetic acid
FBGC	Foreign body giant cells
